# The visceral larva migrans caused by *Toxocara canis*: a case report

**DOI:** 10.11604/pamj.2020.36.150.24176

**Published:** 2020-07-03

**Authors:** Labretesche Gracia Christ Gakosso, Fatima Baadi, Fatima Zahra Abakka, Dounia Basraoui, Hicham Jalal

**Affiliations:** 1 Radiology Department, Mother and Child Hospital, CHU Mohammed VI, Université Cadi Ayyad, Marrakech, Morocco

**Keywords:** Toxocariasis, digestive, imagery

## Abstract

Hepatic toxocarosis is caused by the dog´s roundworm, Toxocara canis. Responsible for an eosinophilic inflammatory syndrome causing liver damage that can be detected on ultrasound, computed tomography and sometimes magnetic resonance imaging. We report the case of a nine-year-old child, living in countryside, with a notion of cohabitation with canids. He presented a digestive symptomatology revealed by abdominal pain, with a hemeosinophilia in the hemogram. The etiological assessment of hyper eosinophilia objectified a positive Toxocara canisserology. The imaging assessment in search of digestive visceral lesions, found multiple heterogeneous hypoechogenic areas, poorly defined, scattered in the liver. On the abdominal CT scan, its areas appear of unenhanced density and low density and better visible after injection of contrast product. This observation reveals that imagery, although not very specific, helps in the assessment of liver damage from digestive toxocarosis.

## Introduction

Helminths and protozoa can determine various digestive manifestations, by the presence of the parasite in the digestive tract. He is the cause of the visceral larva migrans syndrome testifying to the migration of second stage nematode larvae through the tissue of the human viscera. Toxocarosis is a zoonosis corresponding to the infestation of humans by larvae of roundworms belonging to the genus *Toxocara*: it is a parasitic deadlock [1-3]. Cosmopolitan, human contamination occurs less through contact with parasitized animals, than through geophagy, favored by contact with the soil of children who have not acquired standing and handling or consuming contaminated food. Toxocarosis is more common in children than adults, in rural areas than in cities. In adults, the infection is rare and often latent [4]. Two main clinical forms are generally described: visceral toxocariasis and ocular toxocariasis [5]. We report the case of a collected digestive toxocariasis; in the medical imaging department of the Mohammed VI University Hospital Center. Our aim is to illustrate the contribution of imagery in the lesion assessment of liver damage in this condition.

## Patient and observation

**Figure 1:**
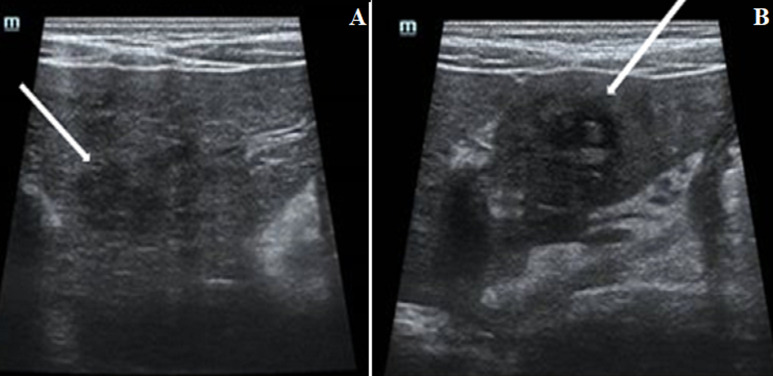
ultrasound images of the liver showing poorly defined heterogeneous hypoechoic areas of variable shape (white arrow), visible in segments IV (A) and III (B) of the liver

**Figure 2:**
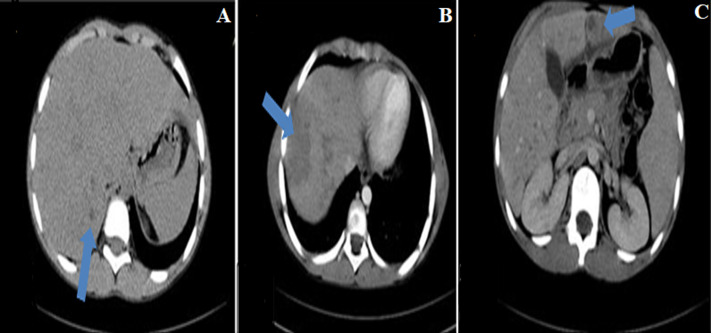
computed tomography images with and after injection of contrast product illustrating the areas (blue arrow) little visible in spontaneous contrast (A), better described after injection of contrast product, scattered in the liver at arterial (B) and portal times (C)

He is a nine-year-old patient, living in a rural area, with no particular pathological history. He consulted in hematology for subacute, poorly systemic abdominal pain of moderate intensity, without notion of digestive disorders, evolving for 15 days, in an apyretic context without alteration of the general condition. The physical examination noted hepatomegaly, with a slight helpless peri-umbilical pain without dermatological lesion or neurological disorders. The hemogram showed an increase in leukocytes at 34,400/μL, with increased eosinophilia at 23,000/μL, motivating the etiological research of the increase of eosinophils, through the realization of a thoracic radiography and an examination of the stools, not finding anomalies. Only the *toxocara*serology was positive with 43 μL of *Toxocara canis* IgG, with confirmation of the presence of *anti-toxocara canis* antibodies. An abdominal ultrasound (Figure 1), performed as part of the lesion assessment objectified the presence of multiple heterogeneous hypoechogenic areas scattered all over the liver. The largest of which was localized at the level of segment VIII, associated with hepatic peri-hilar lymphadenopathy and peri-splenic. On color Doppler, these areas were poorly vascularized. The complementary abdominal CT scan (Figure 2); allowed to better characterize the lesions of the liver, by finding multiple scattered lesions, badly limited on acquisitions not injected, not enhanced and better seen after injection of contrast product at arterial and venous times; crossed by the portal branches, consistent with the radiological characteristics of liver damage by the *larva migrans*digestive *toxocara*. The child was started on albendazol tablet therapy, but was non-observant.

## Discussion

Toxocariasis is known to be a common disease in preschoolers because they play with canines, especially dogs, on clean grounds and are therefore more likely to ingest soil contaminated with *toxocara*eggs. In adults, cases of hepatic toxocariasis have been recently reported and this tendency is so much to be attributed to the particular habit of adults to want to eat undercooked meat [1-4, 6]. In our patient, we found a notion of contact with dogs, which could be the cause of the infestation. The majority of patients are asymptomatic, the disease is generally discovered during the investigation of peripheral eosinophilia [6] and after when the parasite load is high, the patient complains of abdominal discomfort, fever and fatigue. Hepatosplenomegaly can occur [2-4, 6]. When the lungs are involved, patients complain of cough and exertional dyspnea [5]. Biologically, hyper eosinophilia is constantly found in most patients. An enzyme linked immunosorbent test is used for diagnosis. This test is used to detect human IgG antibodies against *Toxocara*anti-excretors/secretors [1]. These clinical and biological data found in the literature, are in perfect adequacy with those found in our patient.

Digestive lesions due to *toxocara* on imaging reflect the granulomas or abscesses surrounding live or dead larvae and or eosinophilic inflammation of the liver parenchyma [1-4, 6-8]. On ultrasound, these lesions described above, correspond to multiple hypo echogenic or mixed echogeneity areas in the hepatic parenchyma, focal or diffuse, of blurred contours, which may be confluent, most often associated with hepatic and mesenteric hilar lymphadenopathies [1, 6-7]. The hepatic echography of our patient found heterogeneous hepatomegaly, which was the site of multiple areas, of mixed echogenicity, poorly vascularized with color doppler, associated with hepatic and splenic peri-hilar lymphadenopathies. Which agrees with the data described in the literature. In the CT scan, these hepatic lesions are translated by multiple iso or hypodense focal zones in spontaneous contrast, ill-defined, let´s take a trapezoid or triangular shape enhancing at the periphery at arterial time, with homogenization at venous time. Which are these lesions are crossed by the segmental branches of the portal trunk. This last acquisition being considered as the phase where the lesions are better visible and can be analyzed [4-7].

This description of the semiology on the CT scan of the hepatic lesions of toxocarosis, was found in our patient with the CT scan complement. Jae Houm Lim *et al*. in South Korea [1], performed complementary magnetic resonance imaging in addition to ultrasound and abdominal computed tomography to better typify lesions. They noted on the sequences without injection, lesions in ranges or in the form of nodules badly limited in hypo signal on the T1-weighted sequences and hypo or hypersignal in T2 and after gadolinium injection, these lesions appeared in the form of ill-defined ranges in hypo signal in portal phase and in iso signal in arterial phase with enhancement in the periphery compared to eosinophilic granulomas. Generally, treatment of *Toxocara sp*.uses anthelmintics [6-8].

## Conclusion

Liver damage due to *Toxocara canis larva migrans syndrome*; result in imaging, by the presence of multiple, ill-defined ranges of variable texture, depending on the type of imaging used, in relation to inflammatory to eosinophilic granulomas. It is therefore important that any radiologist masters these types of lesions so as not to ignore them.
